# Supplementary material for: Infectious Bronchitis Virus Activates the Aryl Hydrocarbon Receptor During In Vitro Infection
Source: Vet Sci. 2025 Sep 24;12(10):932. doi: 10.3390/vetsci12100932 (PMC12567813; doi:10.3390/vetsci12100932)
Supplement: Supplementary file 1 [file vetsci-12-00932-s001.zip › vetsci-3832876-supplementary.pdf]

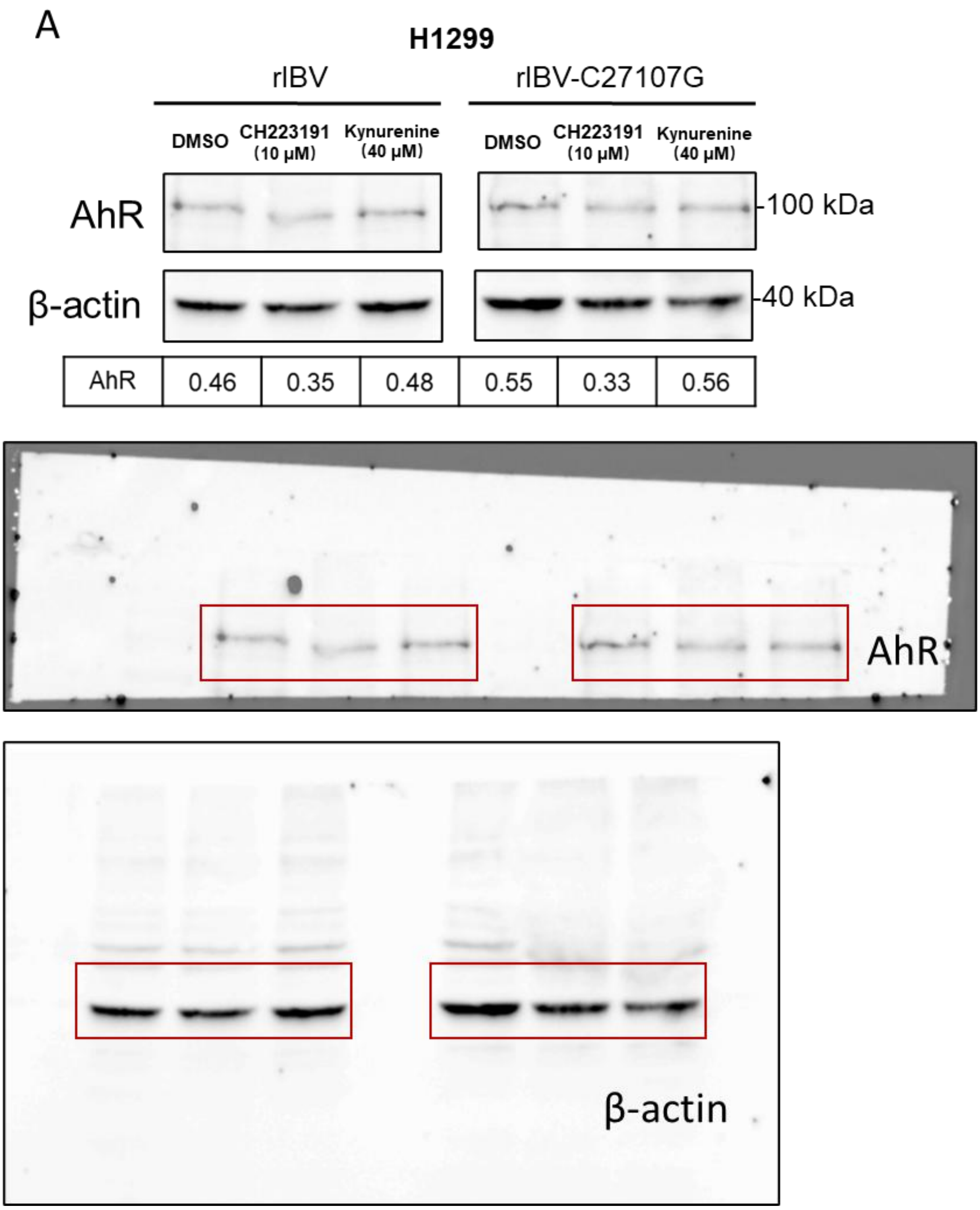

Figure 3. A

Uncropped Western blotting images. AhR and β-actin protein (Marked with red square).

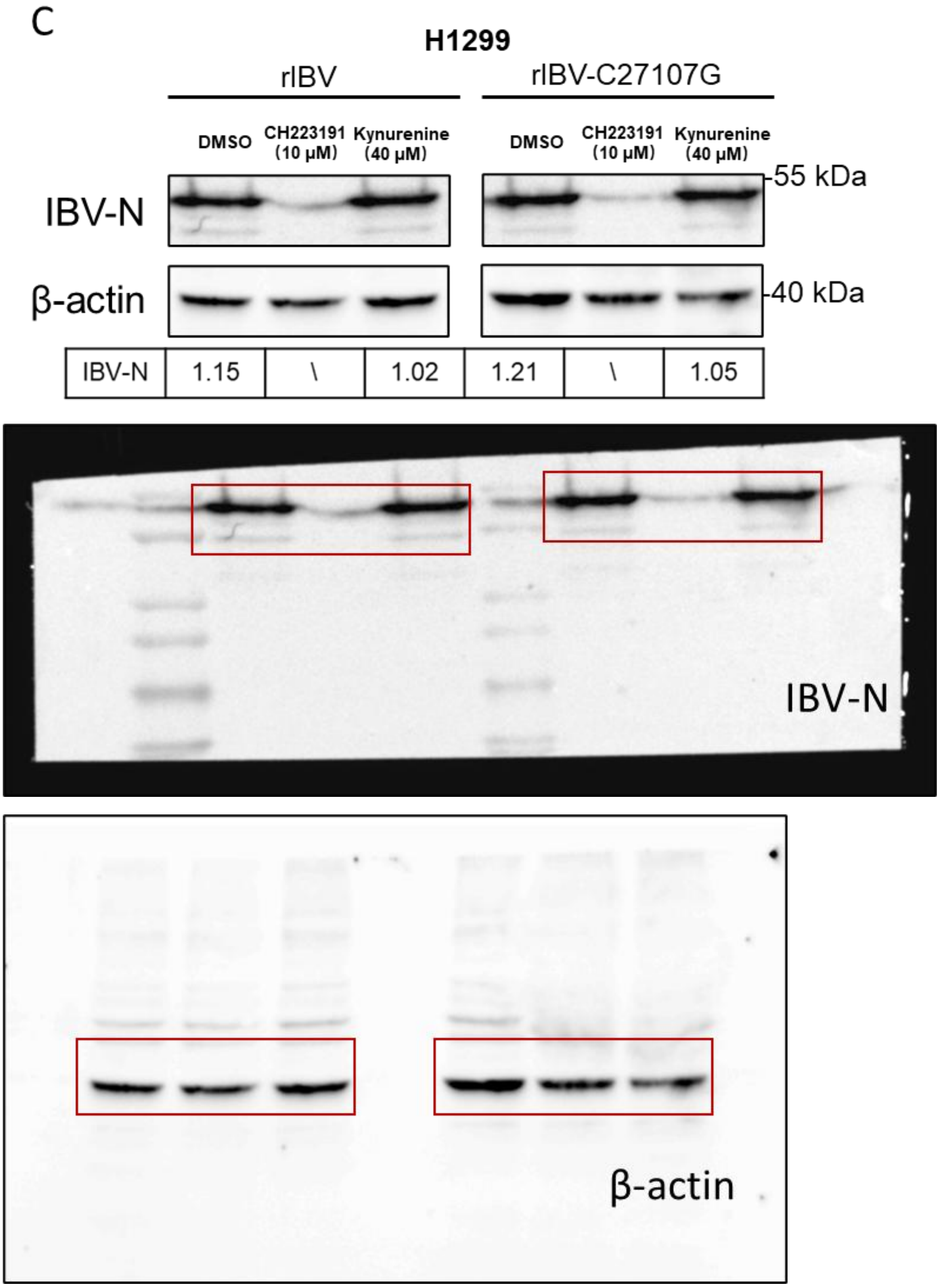

Figure 3. C

Uncropped Western blotting images. IBV-N and β-actin protein (Marked with red square).

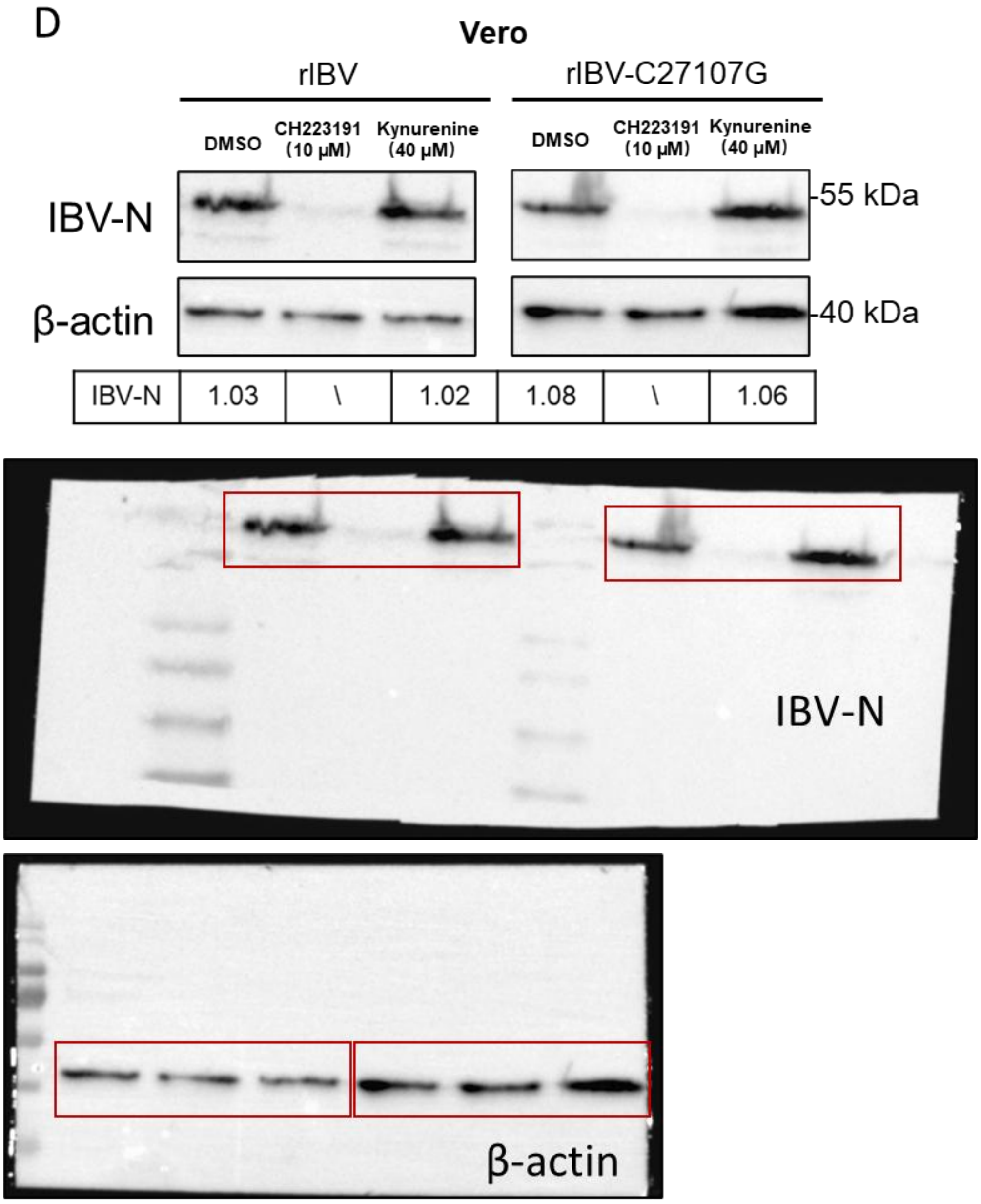

Figure 3. D

Uncropped Western blotting images. IBV-N and β-actin protein (Marked with red square).

B

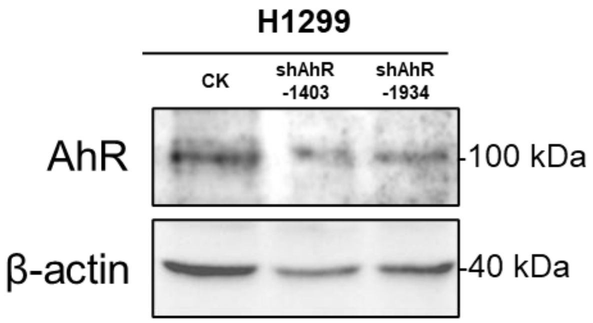

AhR	0.87	0.73	0.96

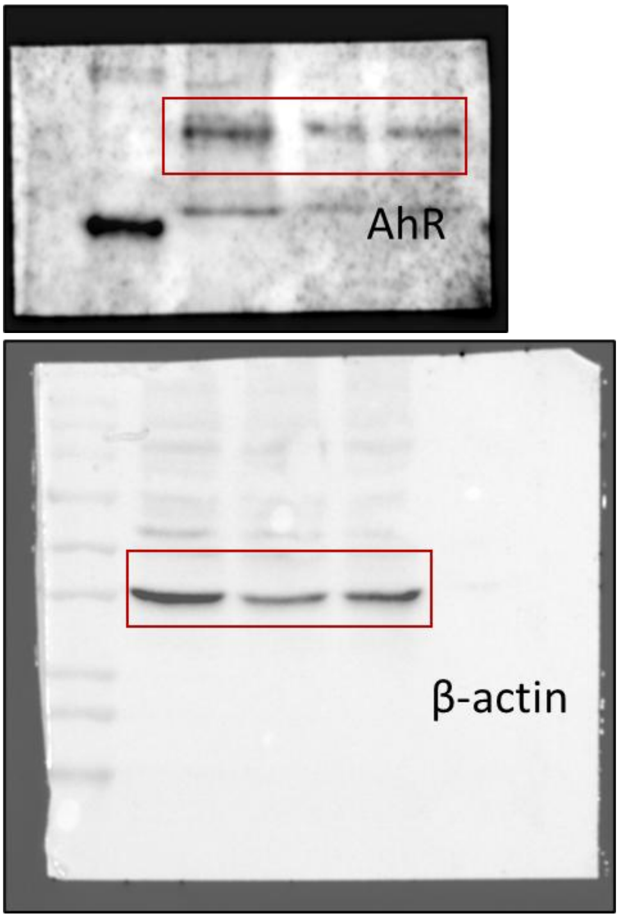

Figure 5. B

Uncropped Western blotting images. AhR and β-actin protein (Marked with red square).

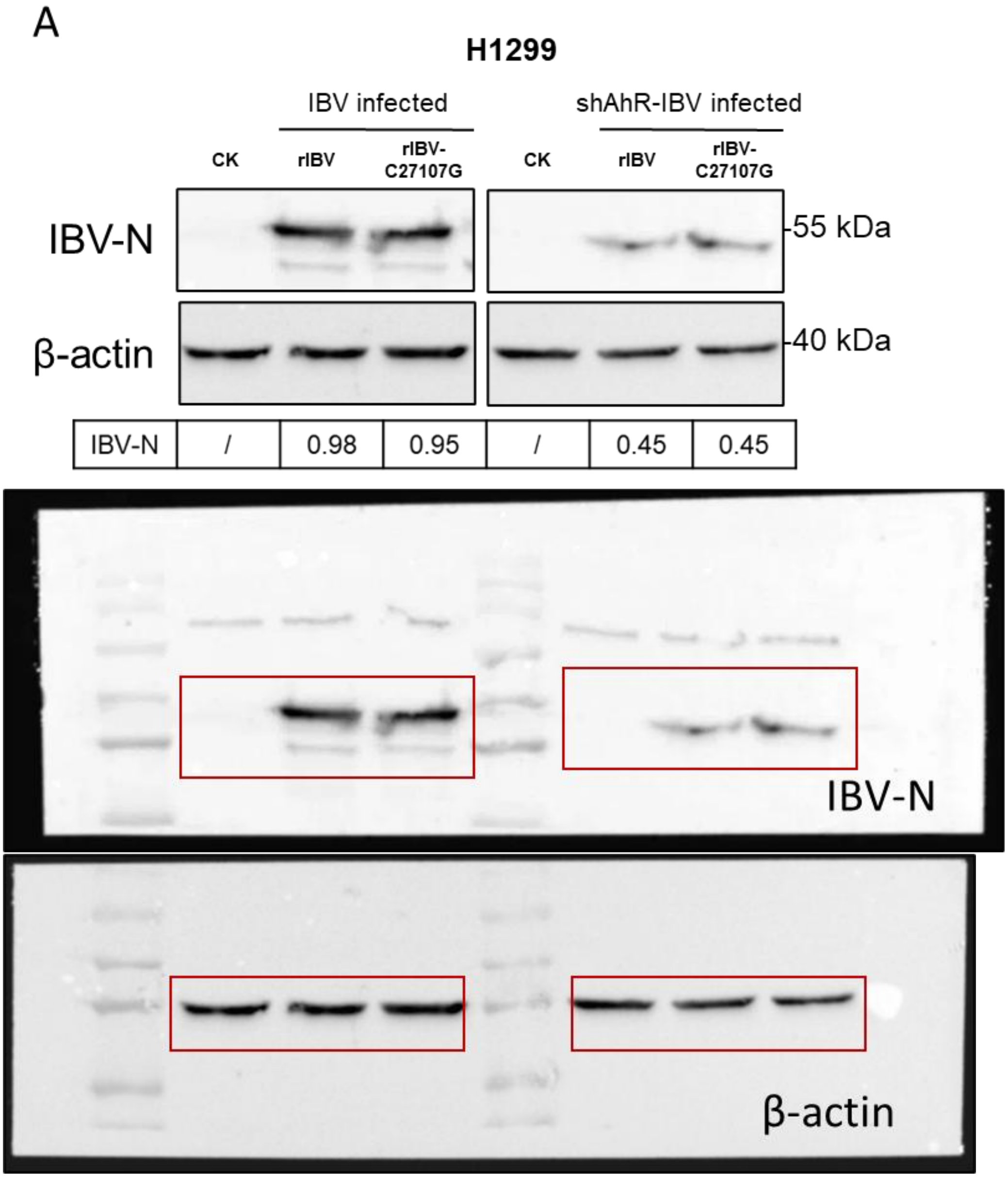

Figure 6.A

Uncropped Western blotting images. IBV-N and β-actin protein (Marked with red square).
